# Morphometric characteristics of the sphenoid sinus and potential influencing factors: a retrospective assessment using cone beam computed tomography (CBCT)

**DOI:** 10.1007/s12565-021-00622-x

**Published:** 2021-07-07

**Authors:** Pradeep Singh, Kuofeng Hung, Deepal Haresh Ajmera, Andy Wai Kan Yeung, Thomas von Arx, Michael M. Bornstein

**Affiliations:** 1 Applied Oral Sciences and Community Dental Care, Faculty of Dentistry, The University of Hong Kong, Hong Kong SAR, China; 2 Oral and Maxillofacial Radiology, Applied Oral Sciences and Community Dental Care, Faculty of Dentistry, The University of Hong Kong, Hong Kong SAR, China; 3 Orthodontics, Faculty of Dentistry, The University of Hong Kong, Hong Kong SAR, China; 4 Department of Oral Surgery and Stomatology, School of Dental Medicine, University of Bern, Bern, Switzerland; 5 Department of Oral Health & Medicine, University Center for Dental Medicine Basel UZB, University of Basel, Mattenstrasse 40, 4058 Basel, Switzerland

**Keywords:** Sphenoid sinus, Extension, Volume, 3-Dimensional, CBCT

## Abstract

The present study aimed to evaluate the morphological characteristics of the sphenoid sinus (SS), and the impact of potential influencing factors on the morphometric features using CBCT imaging. CBCT scans of 148 patients, aged between 15 and 85 (32.88 ± 15.33) years were retrospectively evaluated. DICOM files from the CBCT scans were imported into semi-automatic software and the SS of each patient was assessed for the morphological characteristics including configuration, symmetry, extension, shape, septation, volume, and maximum diameter. Furthermore, potential influencing factors such as age, gender, side, and sinus condition were analysed. A significant association was observed between sinus extension and age. Septation was also found to be significantly associated with age, gender and sinus condition. Besides, sinus volume was significantly associated with gender and sinus condition. No significant influence of shape and side on the morphometric features was noticed. The average volume and diameter of the SS were 6576.92 ± 3748.12 mm^3^ and 30.48 ± 9.28 mm, respectively. In conclusion, the present findings indicate that age, gender and sinus condition have a significant impact on the morphometric characteristics of the SS. Mature sinuses exhibit a post-sellar extension pattern until middle age. In addition, males, and sinuses with healthy sinus condition have larger volumes compared to females and pathological sinuses.

## Introduction

The sphenoid sinus (SS) is an irregular-shaped cavity (Kinnman 1977), that is the most concealed and inaccessible of all the paranasal air sinuses (PAS) (Wiebracht and Zimmer 2014). Enclosed within the body of the sphenoid bone (Costea et al. 2018), it is connected to the neurocranium and viscerocranium through several skull bones (Ozer et al. 2018). Owing to its close association with vital neurovascular and endocrine structures such as the optic nerve, internal carotid artery, cavernous sinus, and pituitary gland, the SS is considered to be a structure of significant clinical relevance (Fasunla 2012). Due to its anatomical location, it is vital while planning surgical approaches to both intra- and extrasellar pathologies (Oliveira et al. 2017).

First seen radiographically at the age of 2 to 3 years, the SS reaches mature size in adolescence by the age of 12–14 years (Pallanch et al. 2013). Although the average volume of the SS reported in recent studies for an adult male and female is 7.672 to 10.005 ± 5.101 cm^3^ and 7.751 to 7.920 ± 3.176 cm^3^ respectively (Gibelli et al. 2018a, b), it may vary due to factors like age, gender, and race. In this regard, Kim et al. reported that Asians exhibit a greater volume of SS due to larger skulls in comparison to the rest of their body (Kim et al. 2010), whereas an Israeli population showed comparatively smaller SS in a study by Cohen et al. (2018), thereby supporting the existence of racial differences.

The mucous membrane might play a significant role in the anatomical and morphological variability of the SS. Nevertheless, its influence on the normal volume is still unclear. Besides, the diversity in size, shape, and location of the sinus may complicate surgical procedures like microsurgical trans-sphenoidal approach and functional endoscopic sinus surgery (FESS) (Eldan Kapur 2012). Therefore, a comprehensive understanding of the morphometric characteristics of the SS is indispensable for safe surgical procedures.

Recently, volume rendering techniques and 3-dimensional (3D) reconstruction models based on semi-automatic processing of computed tomography (CT) images have enabled the volumetric assessment of the SS. Some CT studies have estimated PAS volumes based on 2-dimensional scans, using different formulas (Yonetsu et al. 2000), while others used 3D models (Park et al. 2010). However, to the best of our knowledge, there are no studies analysing SS configuration and dimensions using CBCT scans. Although the role of age and ethnicity in the volumetric assessment of the SS has been documented earlier, data from an indigenous Asian population is rare. Also, there is still a lack of consensus regarding the influence of age and gender on the morphological characteristics of the SS. Hence, it was aimed to analyse the morphological characteristics of the SS in relation to age, gender, side, and condition of the sinus using CBCT imaging. The present investigation hypothesizes that factors including age, gender, side, and sinus condition may have a significant impact on the morphological findings of the SS.

## Material and methods

The present study was conducted in full accordance with the Declaration of Helsinki 2013 (http://www.wma.net) after obtaining approval from the institutional review board (IRB) of the University of Hong Kong/Hospital Authority Hong Kong West Cluster (approval number UW 19-627).

### Study sample

With reference to a previous study (Gibelli et al. 2018a) and using G*Power (version 3.1.9.2, Kiel University, Germany) with an alpha level of 0.05, a power of 80%, and an effect size of 0.49, a minimum sample of 260 SSs (130 subjects) was considered sufficient. Accordingly, CBCT scans performed between January 2016 and August 2019 using a *ProMax 3D Mid* (Planmeca, Helsinki, Finland) from patients referred to the Diagnostic Imaging clinic at the Prince Phillip Dental Hospital which also houses the Faculty of Dentistry of the University of Hong Kong were collected for the present retrospective study. The scans had been done with the following parameters: 90 kVp, 400 μm voxel size, 9.4 s scan time, and 20 cm × 17 cm field of view. The CBCTs were evaluated based on the following inclusion criteria: (1) male/female patients ≥ 15 years old; (2) SS completely visible on the CBCT scan. The exclusion criteria included: patients with a history of previous surgery or trauma in the region of SS; artefacts (acquisition or patient-related) presenting in the SS region; and nasal or facial neoplasms. In addition, immunodeficient patients or patients with evidence of any craniofacial anomaly were excluded. The medical history of the included patients was searched for demographic data including gender and age at the time of imaging.

### CBCT images analysis

Analysis of CBCT scans was performed on a Philips (Philips, Amsterdam, Netherlands) monitor (223 V, LED), with a resolution of 1920 × 1080 pixels. Data was reconstructed with slices of 0.5 mm thickness and a 0.4 mm voxel size. The SS of each patient was assessed for the morphological measurements including configuration, symmetry, extension, shape, septation; and for potential influencing factors such as age, side, and sinus condition using a propriety software (*ROMEXIS* Version 4.4.0.R, Planmeca, Helsinki, Finland).

### Morphological characteristics of the SS

**Fig. 1:**
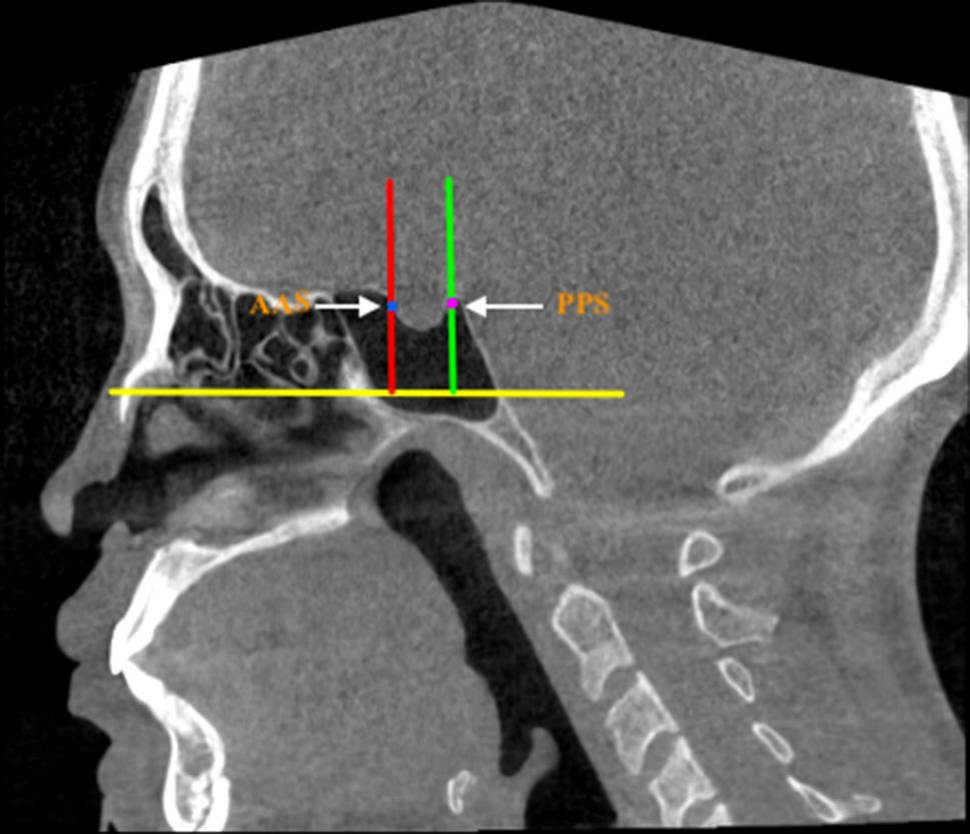
Classification of sphenoid sinus extension based on the boundary of pituitary fossa. AAS (Blue dot)—most anterior point of the anterior wall of sella; PPS (Pink dot)—the most posterior point of the posterior wall of sella; Red line –passing through AAS the red line represents the anterior boundary of pituitary fossa; Green line – passing through PPS the green line represents the posterior boundary of pituitary fossa; Yellow line–Frankfurt Horizontal Plane (FH Plane)

**Fig. 2:**
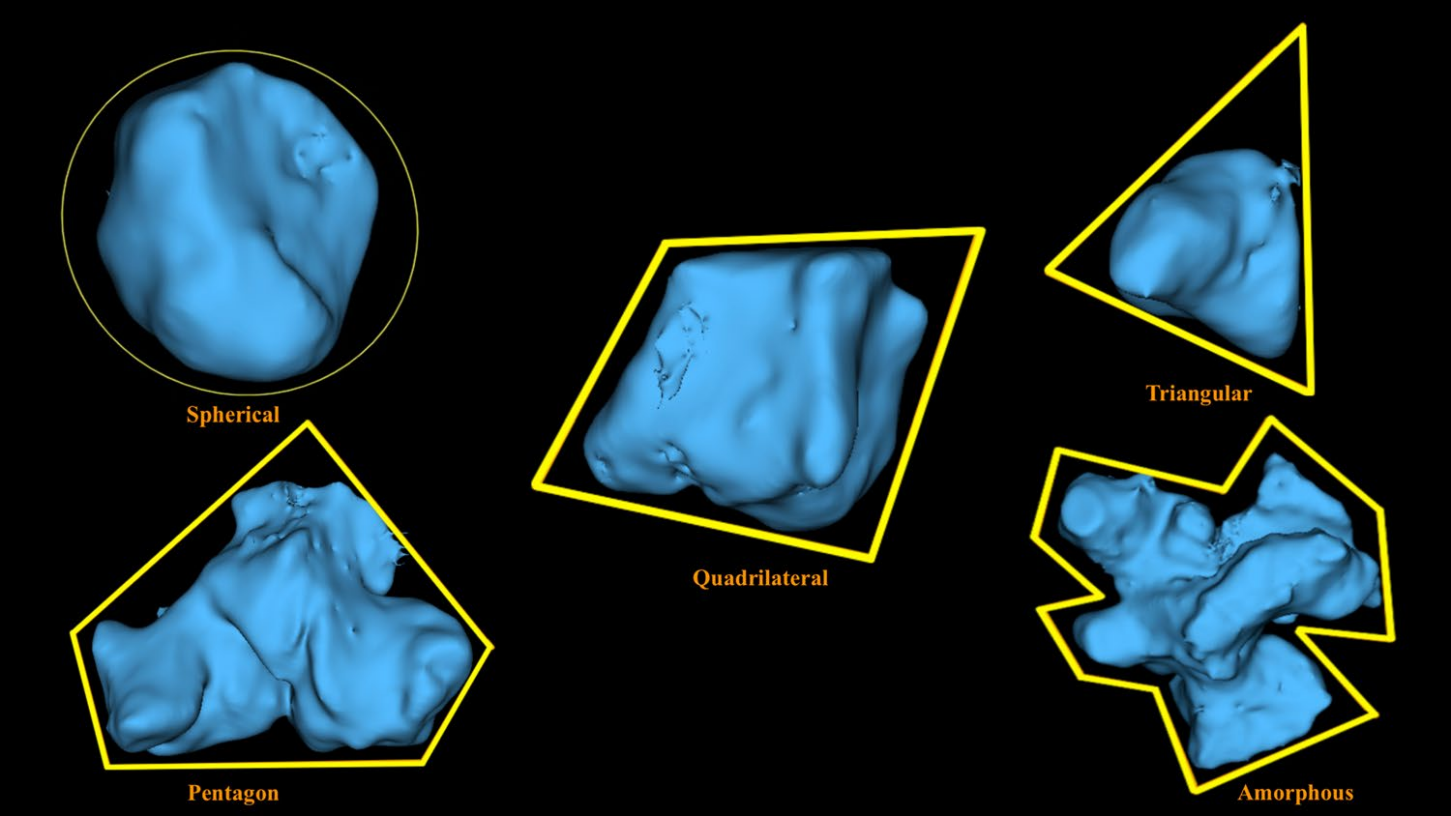
3D reconstructed images of unilateral sphenoid sinuses showing various shapes observed in the coronal view

**Fig. 3:**
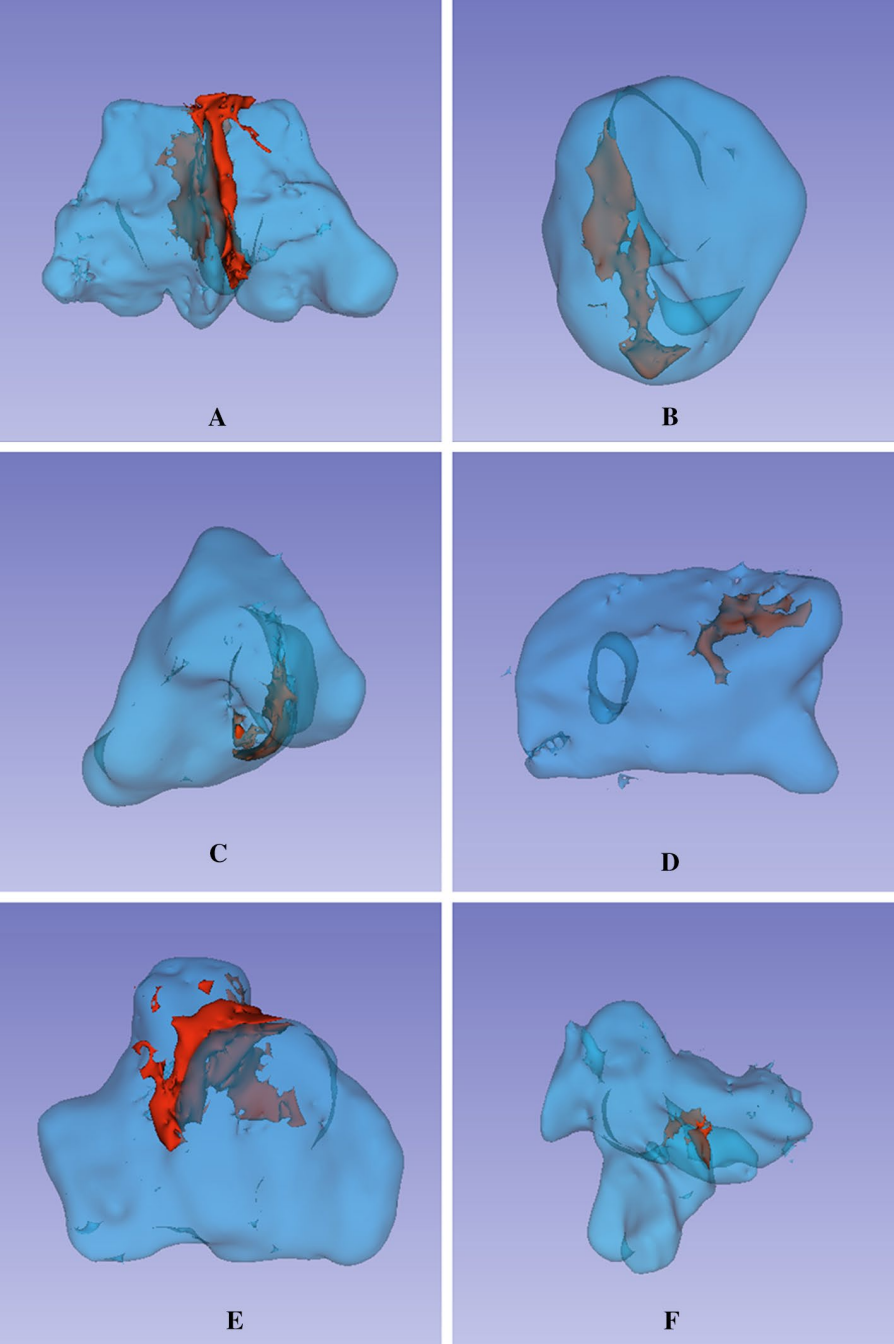
3D reconstructed images of sphenoid sinuses showing inter-sinus and intra-sinus septae (Red) within various sinus shapes (Blue). Bilateral sphenoid sinus with inter-sinus septa (**A**). Figures B to F represent unilateral sphenoid sinuses with intra-sinus spetae. Spherical (**B**); Triangular (**C**); Quadrilateral (**D**); Pentagon (**E**); and Amorphous (**F**)

The details of the definitions, classifications, and abbreviations used in the present study are summarized in Table 1. For the present study, the morphological characteristics of the SS were recorded and evaluated based on the following classifications:

1. Symmetry: according to the location and deviation (whether deviated or non-deviated) of inter-sinus septa in the axial view, the sinus was classified as symmetrical or asymmetrical,
2. Configuration: on the basis of presence or absence of inter-sinus septa, the configuration was classified into solitary, paired, and compound. For compound SS, the largest inter-sinus septum was considered as the primary/main inter-sinus septum,
3. Extension: regarding the type of extension two anatomical landmarks namely: (1) the most anterior point of the anterior wall of sella (AAS) and (2) the most posterior point of the posterior wall of sella (PPS) were manually digitized (Tepedino et al. 2019). Following the classification proposed by Yamashita et al. (2014) two tangents passing through the aforementioned landmarks and perpendicular to FH plane (Frankfurt Horizontal plane) (Pittayapat et al. 2017) were drawn. 1st Line—representing the anterior boundary of the pituitary fossa and 2nd line—representing the posterior boundary of the pituitary fossa (Fig. 1). Accordingly, the extension type was classified as conchal, presellar, sellar, and postsellar.
4. Shape: based on the shape of the 3D reconstructed model of the air cavity (Kim et al. 2013a) in the coronal view, the sinus was categorized into well defined and amorphous. Well defined shapes included: spherical, triangular, quadrilateral and pentagon (Figs. 2 and 3).
5. Septation: based on the number of intra-sinus septa present, the sinus was classified as—without septum, single septum, double septa, and multiple septa (Fig. 3).

**Table 1:** Abbreviations and description of the variables used in the present study

Parameters	Classification	Definition
Symmetry	Symmetrical	Inter-sinus septum is in the middle and non-deviated
	Asymmetrical	Inter-sinus septum is not in the middle and deviated towards right or left side
Configuration	Solitary	Absence of inter-sinus septum
	Paired	Presence of single inter-sinus septum
	Compound	Presence of more than one inter-sinus septum
Extension	Conchal	Slightly pneumatized, small sinus, not related to the sella turcica
	Presellar	Pneumatization does not extend beyond the 1st line
	Sellar	Pneumatization reaches the 2nd line but not extending beyond 2nd line
	Postsellar	Pneumatization crosses the 2nd line and extends beyond the sella turcica
Shape	Well defined	Spherical/Triangular/Quadrilateral/Pentagon
	Amorphous	
Septation	Without septum	Intra-sinus septum absent
	Single septum	One intra-sinus septum present
	Double septa	Two intra-sinus septa present
	Multiple septa	More than two intra-sinus septa present
Age		Patients were divided into four age groups, namely 15–29 years, 30–44 years, 45–59 years and > 60 years
Gender	Male and Female	
Side	Paired sinus	Presence of sinus on the left/right side of inter-sinus septum
	Compound sinus	Presence of sinus on the left/right side of the primary inter-sinus septum
Sinus condition	Healthy	No radiological pathology detected
	Pathological	Presence of membrane thickening, thickening of membrane or complete opacification of sinus

Abbreviations	
SS	Sphenoid sinus
AAS	The most anterior point of the anterior wall of sella
PPS	The most posterior point of the posterior wall of sella
FH plane	Plane passing through the upper border of external auditory meatus upto inferior border of orbital rim

**Fig. 4:**
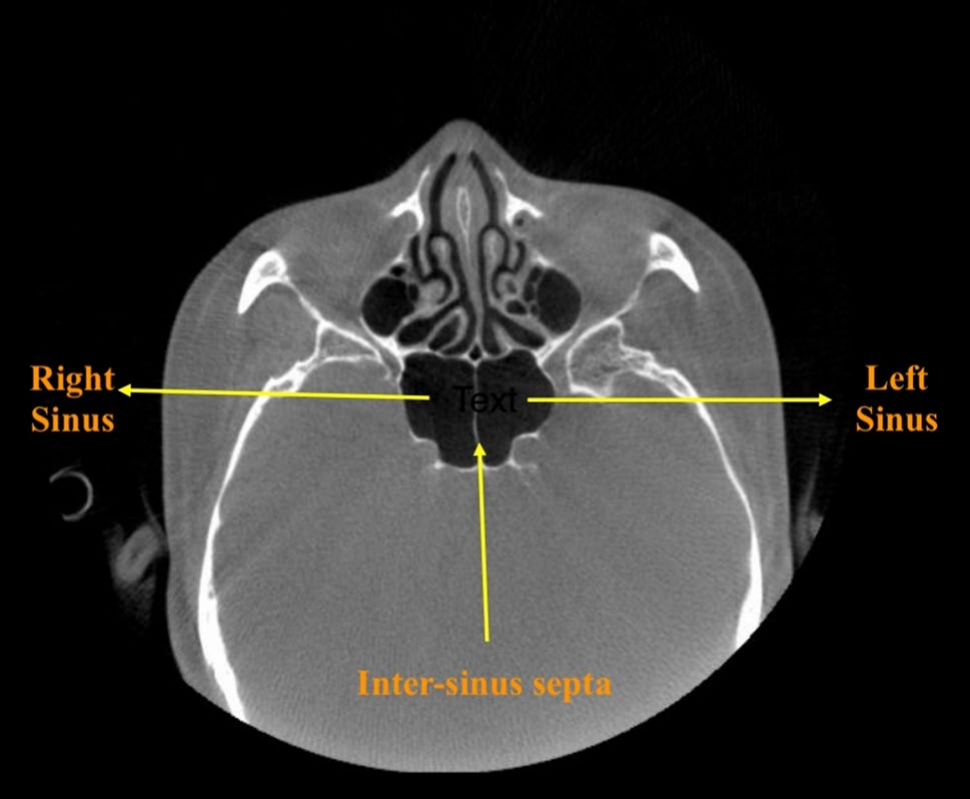
Representative image depicting inter-sinus septum and the left and right sides of a SS

Likewise, the following classifications were used for the recording and evaluation of potential influencing factors (Table 1):

1. Age: patients were divided into four age groups, namely 15–29 years, 30–44 years, 45–59 years and > 60 years,
2. Gender: male and female,
3. Side: side was decided based on the presence of sinus on the right/left side of the primary inter-sinus septum (Figs. 3 and 4),
4. Sinus condition: based on the presence or absence of any pathological finding, the condition of the SS was considered as healthy or pathological. The most common and easily identifiable radiographic features such as membrane thickening, bulging, or opacification were selected for the analysis of sinus condition.

### Dimensional analysis of the SS

For the volumetric analyses, the CBCT scans were imported as DICOM (Digital Imaging and Communications in Medicine) files into *3D Slicer 4.10* (http://www.slicer.org) (Fedorov et al. 2012), installed on a Dell OptiPlex 9010 Desktop (Dell, Round Rock, Texas, USA) with an 18.5-in Dell LCD monitor (resolution of 1366 × 768 pixels; Dell, Round Rock, Texas, USA). The 3D model of the acquired SS was coloured in blue. The sinus volume was determined as an integral volume of the air cavity within the bony walls of the sinus in the sphenoid bone on the reformatted axial, sagittal, and coronal images. In the case of compound SS, the sinus exhibiting the highest dimensional values [maximum volume (mm^3^) and largest diameter (mm)] was recorded for further analysis.

For the assessment of metrical characteristics of the SS, the segmentation and measurements were performed semi-automatically as follows:

1. Manually defining the borders of the SS and cropping the volume of the selected region,
2. Manually marking seeds in the SS cavity on multiple selected sagittal, coronal, and axial slices, respectively,
3. Manually drawing the boundary of the sinus cavity (air cavity within the bony walls of the sinus) in three dimensions (on sagittal, coronal, and axial slices, respectively),
4. Automatic segmentation of the sinus cavity based on the marked seeds,
5. Automatic measurement of the volume (in mm^3^) and maximum diameter (in mm) of each SS including solitary, paired (right and left sinus individually) and compound SS by the software. For compound SS, right and left sinuses exhibiting the highest dimensional values on either side of the primary inter-sinus septum were considered.

### Reliability analysis

To test for inter-observer reproducibility, all observations for categorical variables (age, gender, configuration, symmetry, extension, septation, side, and sinus condition) were performed by two examiners (P.S and K.H). Furthermore, the measurements using continuous variables (volume, and maximum diameter) and shape analysis were performed by one examiner (P.S). After a washout period of 1 month, the same examiner (P.S) performed the measurements and shape analysis again to test for intra-examiner repeatability. Inconsistent findings were resolved by discussion with an expert in the dento-maxillofacial radiology (MB). Only the agreed data was utilized for further reporting and analysis.

### Statistical analysis

First, the intra-class correlation coefficient (ICC) and Cohen’s kappa (*k*) values were determined for intra-observer repeatability and inter-observer reproducibility. Next, the data was analysed descriptively and the frequency of morphological parameters was expressed as counts and percentages. The association between potential influencing factors (age, gender, side, and sinus condition) and the morphometric characteristics of the sinus was estimated using ‘Chi-squared test’ or ‘Fisher-Freeman-Halton exact test’ (if > 20% of the cells have expected count less than 5). Due to non-normality of volume and diameter, the volume and diameter data was log-transformed (log10) before further analyses. Independent samples ‘t-test’ or ‘One-way ANOVA’ (One-way analysis of variance) were performed to explore the association between potential influencing factors (age, gender, side, and sinus condition) and the log-transformed volume and diameter. Further, adjusted models were performed for the variables that showed significant association in the initial analysis. The strength of the association between potential influencing factors and morphometric characteristics was evaluated by their ‘*P*’ values using ‘Logistic regression (Binary or Multi-nominal)’ or ‘Multi-Way ANOVA’ (Multi-Way analysis of variance). The regression models, adjusted for age, gender and sinus condition allowed us to compare the independent effect of various categories of potential influencing factors. For the categorical variables with less observed frequency, categories were merged and a reduced model was used, while ‘Multi-Way ANOVA’ was performed for log-transformed continuous outcomes (volume and diameter).

A significance level (*p)* of < 0.05 was considered for all the tests. Data analysis was performed using SPSS (Version 25.0, IBM Corp., Armonk, NY, USA).

## Results

All measurements were found to be highly reproducible with an excellent intra-observer repeatability and inter-observer reproducibility for observations (configuration, symmetry, extension, shape, septation, side, and sinus condition) and measurements (volume and maximum diameter). Overall, the intra-class correlation coefficient (ICC) and Cohen’s kappa (*k*) values ranged between 0.876–0.999 and 0.931–1.000 respectively for the two observations and measurements.

### General characteristics of the SS parameters

**Fig. 5:**
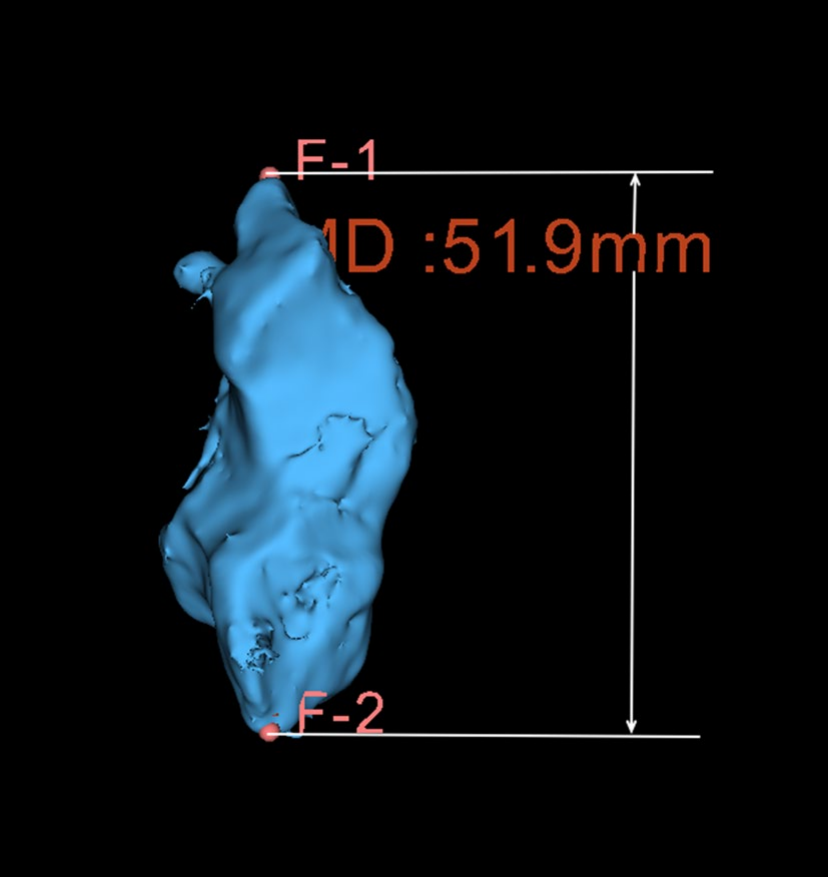
Representative image of a maximum diameter measurement on a volume rendered sphenoid sinus (MD, maximum diameter; F1,F2, fiducial markers)

CBCT scans of 149 patients (72 males and 77 females) aged between 15–85 (32.88 ± 15.33) years were reviewed (Table 2). One male patient showed a complete absence of SS and was excluded from further analysis. Therefore, the final sample consisted of 148 patients (285 sinuses; 11 solitary, 135 paired, and 2 compound). A majority of the sinuses observed in the present study were asymmetrical (85.1%) and presented healthy sinus condition (81.8%). With regard to pathological status, findings such as membrane thickening, membrane bulging or complete sinus opacification, were apparent in 12.6%, 4.2% and 1.4% of the cases, respectively. The morphometric characteristics including shape, volume, and diameter were obtained from a total of 285 reconstructed 3D models. The mean volume of SS observed in the present study was 6576.92 mm^3^ (range 474.89–20620.33 mm^3^; Table 2) while the mean diameter was 30.48 mm (range 16.40–59.5 mm; Table 2; Fig. 5). Slightly higher mean volumes and diameters were noticed for males compared to females (7251.54 mm^3^ and 31.33 mm versus 5943.61 mm^3^ and 29.78 mm, respectively).

**Table 2:** Distribution of morphometric characteristics and potential influencing factors according to gender

Parameters		Male	Female	Total (148 patients)
Age (Mean ± SD) years		33.9 ± 16.3	31.9 ± 14.3	32.9 ± 15.3
Gender		71 (48.0%)	77 (52.0%)	148 (100%)
	Classification	Male	Female	Total (285 sinuses)
Side^δ^	Right	66 (50.0%)	69 (50.0%)	135 (50.0%)
	Left	66 (50.0%)	69 (50.0%)	135 (50.0%)
Sinus condition	Healthy	111 (80.4%)	122 (83%)	233 (81.8%)
	Pathological	27 (19.6%)	25 (17%)	52 (18.2%)
Symmetry	Symmetrical	11 (15.5%)	11(14.3%)	22 (14.9%)
	Asymmetrical	60 (84.5%)	66 (85.7%)	126 (85.1%)
Configuration	Solitary	4 (5.6%)	7 (9.1%)	11 (7.4%)
	Paired	66 (93.0%)	69 (89.6%)	135 (91.2%)
	Compound	1 (1.4%)	1 (1.3%)	2 (1.4%)
Extension	Conchal	2 (1.4%)	0 (0%)	2 (0.7%)
	Presellar	0 (0%)	3 (2.0%)	3 (1.1%)
	Sellar	28 (20.3%)	41 (27.9%)	69 (24.2%)
	Postsellar	108 (78.3%)	103 (70.1%)	211 (74.0%)
Shape	(a) Spherical	33 (23.9%)	39 (26.5%)	72 (25.3%)
1. Well defined	(b) Triangular	23 (16.7%)	28 (19.0%)	51 (17.9%)
	(c) Quadrilateral	56 (40.6%)	63 (42.9%)	119 (41.8%)
	(d) Pentagon	10 (7.2%)	5 (3.4%)	15 (5.2%)
2. Amorphous		16 (11.6%)	12 (8.2%)	28 (9.8%)
Septation	Without septum	26 (18.8%)	31 (21.1%)	57 (20.0%)
	Single septum	48 (34.8%)	66 (44.9%)	114 (40.0%)
	Double septa	41 (29.7%)	36 (24.5%)	77 (27.0%)
	Multiple septa	23 (16.7%)	14 (9.5%)	37 (13.0%)
*Volume (Mean ± SD) mm^3^		7251.5 ± 4126.2	5943.6 ± 3242.9	6576.9 ± 3748.1
^#^Diameter (Mean ± SD) mm		31.3 ± 10.0	29.7 ± 8.5	30.4 ± 9.2

Data expressed in frequency (Percentage) and Mean ± standard deviation

^δ^A total of 270 sinuses (135 paired sinuses) were used for further analysis

*Mean volume of the sphenoid sinus in males, females and total sinuses respectively

^#^Mean diameter of the sphenoid sinus in males, females and total sinuses respectively

### Influence of age, gender, side, and sinus condition on the morphometric features assessed

A statistical analysis of various morphological features such as extension, shape, septation, volume, and maximum diameter with regard to age, gender, side, and sinus condition was performed (Table 3). A significantly higher proportion of post-sellar extension was noticed between age groups 15 to 59 years (*P* < 0.001). Likewise, when morphometric features were analysed in relation to gender, a significantly higher proportion of post-sellar extension was observed among males and females *(P* = 0.042). Besides, males showed significantly higher sinus volume as compared to females (*P* = 0.013). Similarly, when analysed for the condition of the sinus, both the sinus condition groups showed a significantly higher proportion of post-sellar extension as compared to other extension types (*P* = 0.004). In addition, healthy and pathological sinus condition revealed a significantly higher proportion of sinuses with single septum and without septum, respectively (*P* < 0.001), when analysed with regard to septation. Further, healthy sinus condition showed significantly higher sinus volume as compared to pathological sinus condition (*P* = 0.033). However, no significant difference in the morphological features was noticed according to the side of the sinus.

**Table 3:** Association between potential influencing factors and the morphometric characteristics of the sphenoid sinus

		Potential influencing factors													
		Age (years)					Gender			Side			Sinus condition		
		15–29 (*n* = 185)	30–44 (*n* = 45)	45–59 (*n* = 26)	> 60 (*n* = 29)		Male (*n* = 138)	Female (*n* = 147)		Right (*n* = 135)	Left (*n* = 135)		Healthy (*n* = 233)	Pathological (*n* = 52)	
Morphometric characteristics	Classification	% within groups, *p*													
Extension^*χ*^	Conchal	1.1%	0.0%	0.0%	0.0%	< 0.001*	1.4%	0.0%	0.042*	0.7%	0.7%	1.000	0.4%	1.9%	0.004*
	Presellar	1.6%	0.0%	0.0%	0.0%		0.0%	2.0%		1.5%	0.7%		0.0%	5.8%	
	Sellar	18.4%	15.6%	42.3%	58.6%		20.3%	27.9%		23.7%	23.7%		23.6%	26.9%	
	Postsellar	78.9%	84.4%	57.7%	41.4%		78.3%	70.1%		74.1%	74.8%		76.0%	65.4%	
Shape^*χ*^	Spherical	22.2%	31.1%	26.9%	34.5%	0.443	23.9%	26.5%	0.500	25.9%	23.7%	0.286	27.0%	17.3%	0.620
	Triangular	17.8%	20.0%	23.1%	10.3%		16.7%	19.0%		14.8%	23.0%		17.6%	19.2%	
	Quadrilateral	44.3%	33.3%	42.3%	37.9%		40.6%	42.9%		45.2%	39.3%		41.2%	44.2%	
	Pentagon	4.3%	4.4%	3.8%	13.8%		7.2%	3.4%		3.0%	5.9%		5.2%	5.8%	
	Amorphous	11.4%	11.1%	3.8%	3.4%		11.6%	8.2%		11.1%	8.1%		9.0%	13.5%	
Septation^*χ*^	Without septum	20.5%	15.6%	23.1%	20.7%	0.095	18.8%	21.1%	0.138	22.2%	17.0%	0.584	15.0%	42.3%	< 0.001*
	Single septum	45.9%	37.8%	26.9%	17.2%		34.8%	44.9%		37.8%	45.2%		41.2%	34.6%	
	Double septa	21.6%	35.6%	34.6%	41.4%		29.7%	24.5%		27.4%	25.2%		29.2%	17.3%	
	Multiple septa	11.9%	11.1%	15.4%	20.7%		16.7%	9.5%		12.6%	12.6%		14.6%	5.8%	

Median, *p*														
Volume (mm^3^)^*at*^	6284.5	7581.0	6041.9	4381.6	0.800	6561.9	6091.7	0.013*	6182.7	6494.1	0.996	6694.1	4486.0	0.033*
Diameter (mm)^*at*^	27.8	29.1	26.1	27.1	0.119	28.3	27.4	0.249	27.3	28.1	0.967	27.6	27.7	0.574

*Right*  right sinus, *Left*  left sinus, *χ*   Chi-squared test/Fisher-Freeman-Halton exact test,* at*  One-way Analysis of Variance (ANOVA)/Independent Samples *t* Test for log-transformed (log10) data

*p < 0.05 (in **bold** *italics*), considered statistically significant

Table 4 provides an overview of the association between potential influencing factors and the various morphometric characteristics across various regression models after adjusting for age, gender and sinus condition. The binary logistic regression revealed that the age was significantly associated with extension, specifically the age groups 15–29 and 30–44 years were more likely to develop post-sellar type extension (*p* < 0.001, and < 0.001, respectively) as compared to age groups more than 44 years. Likewise, septation was found to be significantly associated with age (*P* = 0.044) wherein a significantly increased likelihood of sinus with septation was observed among age groups < 60 years (*P* = 0.044). Besides, males presented a significant association with septation (*P* = 0.046). A substantially increased likelihood of septations within the sinus was observed among males, as compared to females. In addition, substantially larger sinus volume was noticed among males (*P* = 0.011) as compared to females. Besides, healthy sinus condition was found to be significantly associated with septation (*P* < 0.001) as compared to pathological sinus condition. In particular, an increased likelihood of sinus with single, double and multiple septations (*p* = 0.001, 0.001 and 0.003 respectively) was noticed within healthy sinus condition. Lastly, healthy sinus condition presented significantly larger SS volume (*P* = 0.006) as compared to pathological sinus condition.

**Table 4:** Regression analysis for the association between potential influencing factors and the morphometric characteristics of the sphenoid sinus

Morphometric characteristics	Potential influencing factors							
	Age				Gender		Sinus condition	
	15–29	30–44	45–59		Male		Healthy	
	p value			*P*	p value	*P*	p value	*P*
Binary Logistic Regression								
Extension								
CPS^‡^								
Postsellar	< 0.001^*^	< 0.001^*^	0.262	** < *0.001***^**†**^		0.176		0.057
Multi-nominal Logistic Regression								
Septation								
Without septum^‡^								
Single septum	0.096	0.136	0.655	***0.044***^**†**^	0.682	***0.046***^**†**^	0.001*	** < *0.001***^**†**^
Double septa	0.249	0.802	0.633		0.197		0.001^*^	
Multiple septa	0.346	0.720	0.543		0.068		0.003^*^	
Multi-way ANOVA								
Volume	0.557	0.362	0.966	0.769		***0.011***^**†**^		***0.006***^**†**^
Diameter	0.230	0.023	0.588	0.114		0.231		0.514

*CPS*  reduced model (Conchal, Presellar and Sellar merged)

^‡^Reference group; Age > 60, female gender, and pathological status were redundant

^**†**^*P* < 0.05 (in **bold** *italics*), considered statistically significant

*p value comparisons between the corresponding individual category and the reference category

## Discussion

A comprehensive understanding of the normal morphology and the role of potential influencing factors is important for accurate diagnosis, knowledge of pathologies, and pre-operative surgical assessment of the SS. The present study confirmed that SS morphology varies across sinus shapes, septation and extension wherein the quadrilateral shape, single septation, and post-sellar type were found to be more frequent. Although extension variability has been discussed in the literature using CT and magnetic resonance imaging techniques, there has been no CBCT based study yet. *Gibelli *et al*.* highlighted a high frequency of the sellar type (74%) in their study (Gibelli et al. 2018b). However, the post-sellar type was found to be most frequent (74.0%) in the present sample, which was in accordance with earlier data from *Öksüzler *et al*.* and *Kim *et al*.* 2013b (Öksüzler et al. 2019). Besides, present findings also revealed that a mature sinus (> 12 years) (Pallanch et al. 2013) might exhibit post-sellar extension until middle age. Further, the frequency of conchal, presellar, and sellar type SS was very low in the present study.

The shape of the SS may be indicative of the progression of extension into the adjacent bone. For instance, the pentagon shape suggests the extent of pneumatization into the greater wing and pterygoid plate of the sphenoid (Rennie et al. 2017)*.* Only one previous study (Rennie et al. 2017) analysed the 3-dimensional forms (3D) of SS in detail. Where *Rennie *et al*.* observed six 3D forms of SS in their study (Rennie et al. 2017), the present study identified five different forms. In the present study, quadrilateral type (41.8%) was found to be the most common shape, while pentagon (5.2%) was the least frequent. These results are in accordance with those of previous data (Rennie et al. 2017). Interestingly, no significant association was noticed between different sinus shapes and potential influencing factors, thereby suggesting that SS shape is independent of age, gender or sinus condition and is inconsistent in right and left sinuses.

The SS is usually divided into paired or compound sinuses by one or more inter-sinus/main septa. In the case of a paired sinus, the septum is often paramedian and deviated laterally (Anusha et al. 2014), thus dividing the sinus into two asymmetric sinuses. The location of the inter-sinus septum in the present study was consistent with the findings of *Rennie *et al*.* and *Anusha *et al*.* (Anusha et al. 2014; Rennie et al. 2017). The inter-sinus septum is considered to be a crucial structure for trans-sphenoidal surgery as it lies in close proximity to the optic nerve and internal carotid artery and sometimes may be attached to the bone covering these vital structures (optic nerve and internal carotid artery) (Famurewa et al. 2018). Therefore, careful consideration is required if inter-sinus septum is planned to be perforated during trans-sphenoidal surgery to avoid inadvertent injury to the optic nerve or to the internal carotid artery. Some studies have identified a solitary or a compound sinus. *Hamid *et al*.* noticed a solitary sinus in 10.8% (Hamid et al. 2008), while *Sareen *et al*.* observed a compound sinus in 80% (Sareen et al. 2005) which was in contrast to the present findings, where a frequency of 7.4% for solitary and 1.4% for compound sinuses was observed. This is an important finding from a surgical point of view, since an increase in the number of inter-sinus septa may also increase the risk of complications. Where *Odat *et al*.* showed a frequency of 52% intra-sinus septa, most of the cases in the *Rennie *et al*.* study were without septum (Odat et al. 2019; Rennie et al. 2017). Noticeably, in the present study, 40.0% of the cases showed a single intra-sinus septum while the frequency of multiple septa and sinus without septum was 13.0% and 20.0%, respectively. *Odat *et al*.* suggested that although the frequency of intra-sinus septa increases with age, the number of intra-sinus septa seems not to be affected by the sinus condition (Odat et al. 2019). In contrast, the present findings showed no significant increase in intra-sinus septations with age. However, some degree of septation (single, double or multiple septa) might always be associated with a mature sinus (> 12 years) (Pallanch et al. 2013) as revealed from the current findings. Also, a healthy sinus condition always exhibited some level of septation in the present study, thereby implying that a sinus without intra-sinus septa might be indicative of pathological sinus condition.

An essential consideration for the decision of sellar region surgery is also the size (including volume and maximum diameter) of the sinus. In the current study, the normal volume and mean maximum diameter recorded were 6576.92 mm^3^ and 30.48 mm, respectively which was in accordance with the findings of *Oliveira *et al. *(*Oliveira et al. 2017*)*. These values were somewhat smaller as compared to the dimensions reported in previous studies (Gibelli et al. 2018a; Öksüzler et al. 2019; Ozer et al. 2018). A reasonable explanation for such contrasting results could be the existence of an ethnic variability, since the aforementioned authors (Gibelli et al. 2018a; Öksüzler et al. 2019; Ozer et al. 2018) conducted their studies in Italian and Turkish subjects. Likewise, *Kim *et al*.* reported larger volumes in Asian subjects of Korean descent (Kim et al. 2010), which was in contrast to the present findings. This disparity could be due to potential racial variability or because of difference in the methodology for SS volume estimation. Further, a decrease in SS width with increasing age has been reported by Öksüzler et al. In contrast, no significant association between age and SS diameter was noticed in the current study. Noticeably, the current findings were suggestive of a larger SS in males as compared to females, and larger SS presented healthy sinus condition more frequently as compared to smaller SS.

SS may undergo several pathological changes that require early detection for timely diagnosis. For instance, SS pathology may manifest radiologically as membrane thickening in conditions such as isolated sphenoid sinusitis, pituitary apoplexy, and anterior/posterior pituitary dysfunction (Arita et al. 2001). This membrane thickening can be the result of a sudden increase in intrasellar pressure which causes congestion of dural blood flow, thus leading to membrane thickening (Takasuna et al. 2017). In the present study, although, the frequency of pathological findings (18.2%) such as membrane bulging or complete sinus opacification was much less compared to the percentage of healthy sinuses (81.8%), their timely diagnosis is paramount. This is emphasized by the fact that partial or complete opacification of the SS may be indicative of a developing pathology such as mucocele, cavernous haemangioma, squamous cell carcinoma, adenoid cystic carcinoma, meningioma, or pituitary macroadenoma (Martin et al. 2002). Hence, due to the thin walls of the SS, such pathologies may disseminate into intracranial structures, leading to fatal complications (Gibelli et al. 2018b).

The findings of the present study will help surgeons, radiologists, and pathologists in the evaluation of normal versus pathological changes of the SS. The multiplicity of anatomical variants may influence surgical access to the hypophysis and associated skull base structures. Therefore, the outcome of a trans-sphenoidal approach is dependent on the surgeon’s anatomical knowledge. Inappropriate forces reaching the cranium caused by too high placement of the nasal septum osteotome into the nasal cavity while performing Le Fort I osteotomy may result in serious bleeding, neurological complications, or even blindness (Saruhan et al. 2018). In the presence of extension, especially the sellar type, which exhibits thinning of the anterior wall, the frequency rate of such complications is high. Therefore, pre-intervention evaluation of extension types is indispensable to avoid iatrogenic injury to the adjacent vital structures or reduce the likelihood of cerebrospinal fluid leakage, which may develop as a repercussion of sinus or orthognathic surgery (Fraederich et al. 2015).

Some limitations of the present study need to be considered. Firstly, the present sample consisted of Asian subjects only, and these findings need to be compared to other populations as well. Secondly, the detailed shape classification mentioned in the present study was based on a subjective grading. The aim was to report that the SS can present well defined as well as amorphous shapes. Since there is no gold standard available to record and report the SS shapes currently, the assessment was standardized by recording the shapes in coronal view only. Finally, the sample size used in the current analysis was relatively small and the data was collected retrospectively. Therefore, the present findings should be interpreted with caution before any generalization of the results.

## Conclusions

The present findings, highlighted the morphological variability across different SS shapes, septation and extension, with quadrilateral shaped sinus, single septation, and post-sellar extension exhibiting a higher frequency. Morphologically, there is no significant difference between the right and left sides of the sinus. Besides, following conclusions can be drawn within the limitations of this study:

- A majority of the sphenoid sinuses were paired, asymmetrical and healthy,
- Mature sinus was significantly associated with post-sellar extension and some degree of septation,
- Males presented a larger septated sinus as compared to females,
- Healthy sinus condition was associated with larger and septated sinus.

## Data Availability

The datasets generated during and/or analysed during the current study are available from the corresponding author on reasonable request.
